# Locally recurrent extraskeletal myxoid chondrosarcoma of the shoulder: a case of complete neoadjuvant radiotherapy response

**DOI:** 10.1186/s13569-020-00150-8

**Published:** 2020-12-11

**Authors:** Luca Improta, Sergio Valeri, Rossana Alloni, Chiara Pagnoni, Francesco Mallozzi Santa Maria, Beniamino Brunetti, Carlo Greco, Irene Aprile, Mirella Maselli, Bruno Vincenzi, Alessandro Gronchi

**Affiliations:** 1 Campus Bio-Medico University of Rome, Via Alvaro del Portillo, 200, 00128 Roma, RM Italy; 2 IRCCS Fondazione Don Carlo Gnocchi ONLUS, Florence, Italy; 3 Fondazione IRCCS Istituto Nazionale Dei Tumori, Via Venezian, 1, 20133 Milan, MI Italy

**Keywords:** Soft tissue sarcoma (STS), Sarcoma referral centers (SRCs), Extraskeletal myxoid chondrosarcoma (EMC), Preoperative radiotherapy, Wide surgical margins

## Abstract

**Background:**

Extraskeletal myxoid chondrosarcoma (EMC) is a rare soft tissue tumor that typically affects the lower limbs of men between the ages of 50 and 60. EMC of the shoulder is rare with a high risk of local recurrence and distant metastasis. A planned surgical excision in sarcoma referral centers (SRCs) is mandatory to obtain the best outcome. The role of chemotherapy (CHT) and Radiotherapy (RT) on soft tissue chondrosarcoma is still controversial.

**Case presentation:**

A 47-year-old man presented to our referral center with a history of EMC in the right shoulder excised with microscopic positive surgical margins in a non-referral center. Staging imaging exams did not reveal distant metastasis or residual disease, but during follow-up a local recurrence was detected. After a multidisciplinary discussion, preoperative radiotherapy was administered with a total dose of 50 Gy, and then the patient underwent wide surgical excision. Histological examination was negative for viable tumor cells. No relapse occurred in a 24-months post-operative follow up.

**Conclusions:**

The case here described suggests the importance of patient’s management in SRCs. A planned combined treatments with both surgery and RT seems to be the best choice to improve local control. RT seems to be promising within this specific histotype. Further studies are needed to confirm if the observed efficacy of combined treatments reflects in a consistent survival benefit for EMC patients.

## Background

Extraskeletal myxoid chondrosarcoma (EMC) is a rare malignant soft tissue tumor with a high risk of local recurrence and metastasis but with an indolent course [1]. Literature data reveal EMC to have an indolent but resilient course with late risk of metastatic progression and local recurrence in the vast majority of patients [2, 3]. Typically, it affects patients between the ages of about 50 and 60 years, more frequently men than women. EMC has a reported overall survival rate at 5 years of 82%, at 10 years of 65%, at 15 years of 58%. The lower limbs are the most common site of EMC while the shoulder is an extremely rare localization [4, 5].The most common site of metastasis is the lung ( more than half of all patients); moreover metastases in deep soft tissue, lymph node, bone and brain have also been reported [3].

According to the ESMO-EURACAN clinical practice guidelines and present literature a localized extremity soft tissue sarcoma (STS) of the adult requires wide surgical excision, preferably associated with radiotherapy (RT) administered both in neo- or adjuvant settings, after a multidisciplinary tumor board discussion and planning [6, 7]. There is no scientific agreement about the effectiveness of chemotherapy (CHT) and RT on soft tissue chondrosarcoma [8–12] and a very small number of cases and studies that may guide local management strategies for patients with EMC; have been reported; for this reason, management of these patients in sarcoma referral centers (SRCs) is strongly recommended since they are associated with a significantly better outcome; therefore each therapeutic decision has to be taken within a dedicated multidisciplinary tumor board (MTB) [7, 13]. Unfortunately, unplanned excision in non-referral centers remains a common problem for STS and exposes patients to a higher risk of residual disease and local recurrence (LR) [14].Patients who underwent unplanned excisions have been found to have an overall risk of local recurrence of 34% instead of a 6% risk of those who underwent planned oncological excisions, with a 5-year estimated local recurrence-free survival of 63.7% versus 89.7% [15]. Residual disease is found in approximately 50% of specimens after systematic re-excision (RE) [16, 17].

We here describe the case of a recurrent EMC of the shoulder, that first underwent an inadequate surgery in a non-referral center and then was treated with preoperative radiotherapy (RT) followed by wide surgical excision and flap reconstruction, showing a complete pathological response to the RT.

## Case presentation

**Fig. 1:**
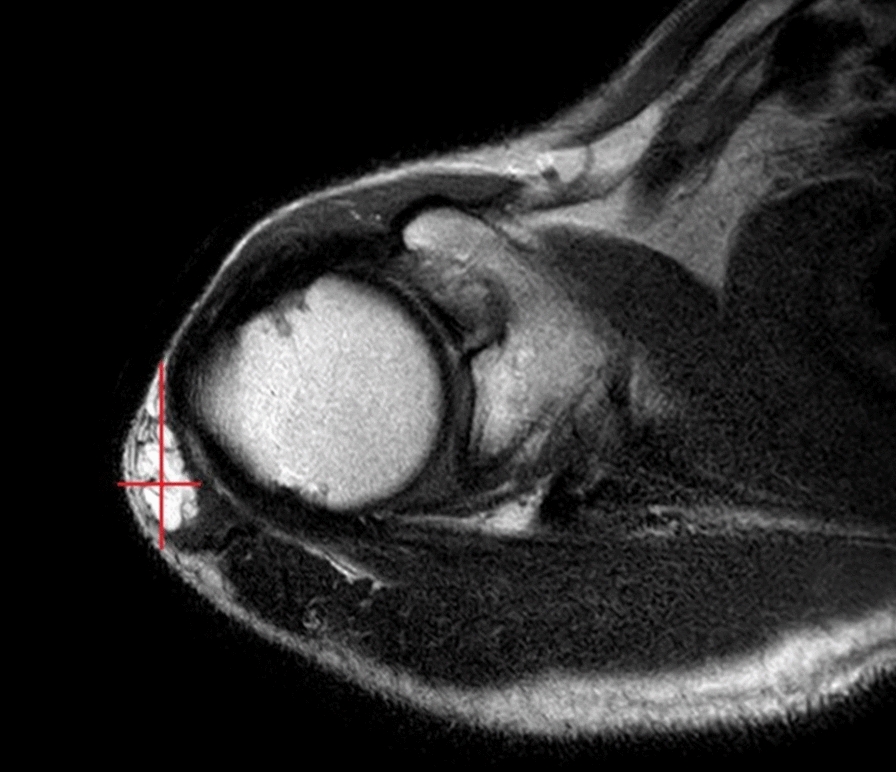
MRI at recurrence presentation, showing a 35 × 15 mm mass within the right deltoid muscle

**Fig. 2:**
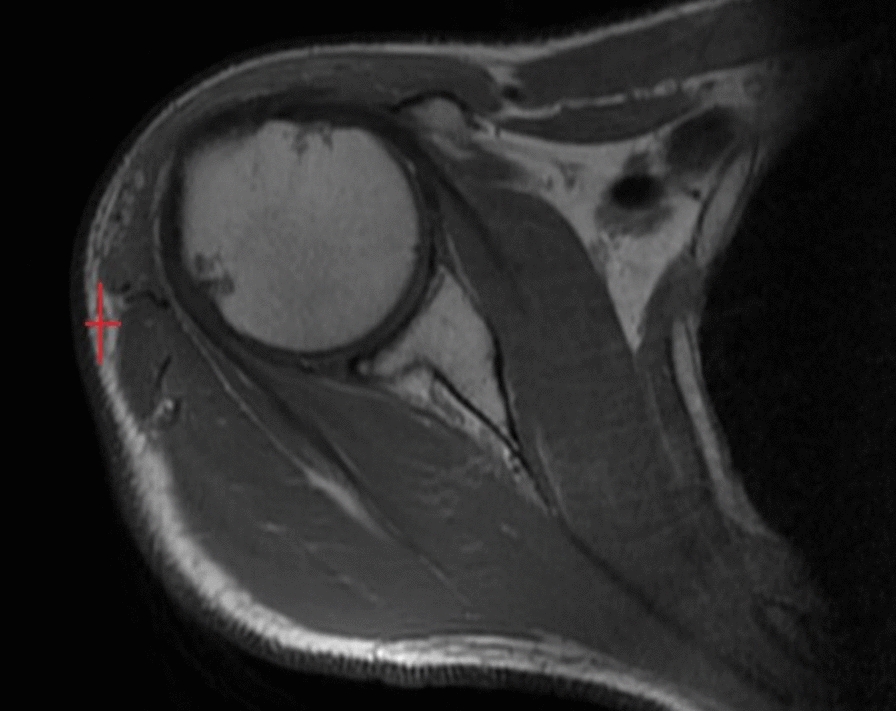
MRI after 3 weeks from RT, showing a partial radiological response, with a mass measuring 16 × 6 mm

A 47-year-old man presented to our referral center in November 2017, with a history of EMC of the right shoulder improperly excised with unplanned microscopic positive surgical margins (R1). The patient underwent debulking of the primary mass in September 2017 in a non-referral center, without a preoperative core-needle biopsy. The histological examination diagnosed EMC. No post-operative treatments were provided. At our first evaluation, the patient did not show macroscopic residual disease or distant metastasis both at the clinical and the radiological assessment. The pathological review of the excised specimen confirmed the diagnosis of EMC. After MTB discussion a “watch and wait” approach was adopted. After a 4-months follow-up, the clinical examination showed an elastic soft painless mass in the right shoulder. A Magnetic Resonance Imaging (MRI) of the shoulder was performed, showing a 35 × 15 mm intramuscular solid mass (Fig. 1) within the right deltoid muscle. Computed Tomography (CT) did not reveal distant metastasis. The case was discussed in our MTB where the decision was made for preoperative RT, with a total dose of 50 Gy (fractionated in 200 cGy/die) which was a administered with Rapidarc technique. Post radiotherapy MRI performed 3 weeks later showed a partial radiological response, with a mass measuring 16 × 6 mm (Fig. 2).

**Fig. 3:**
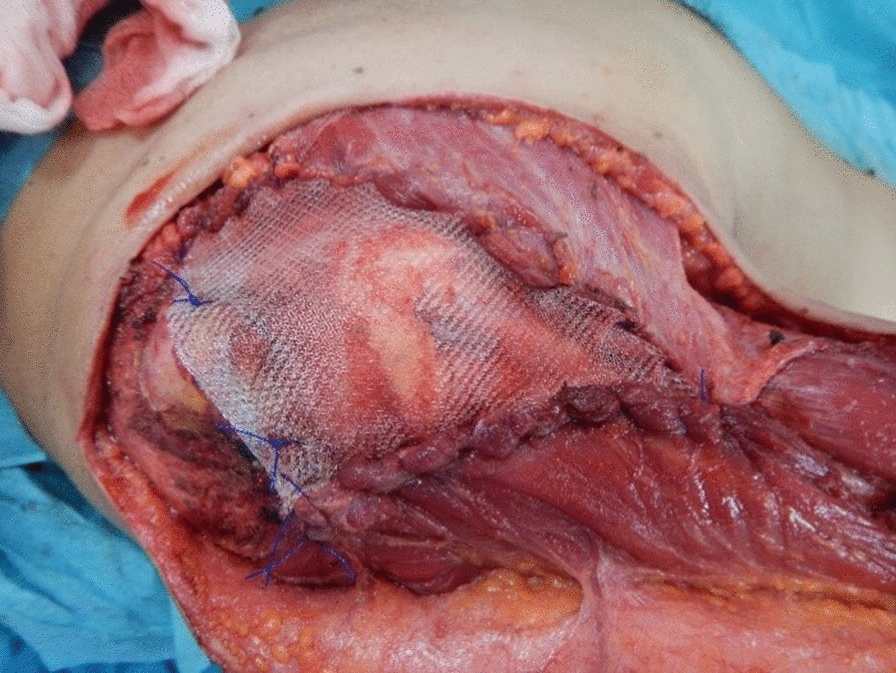
Intraoperative view. After the excision, a polypropylene mesh was placed to reinforce the exposed shoulder joint fixing the deltoid margin to the pectoralis major margin

**Fig. 4:**
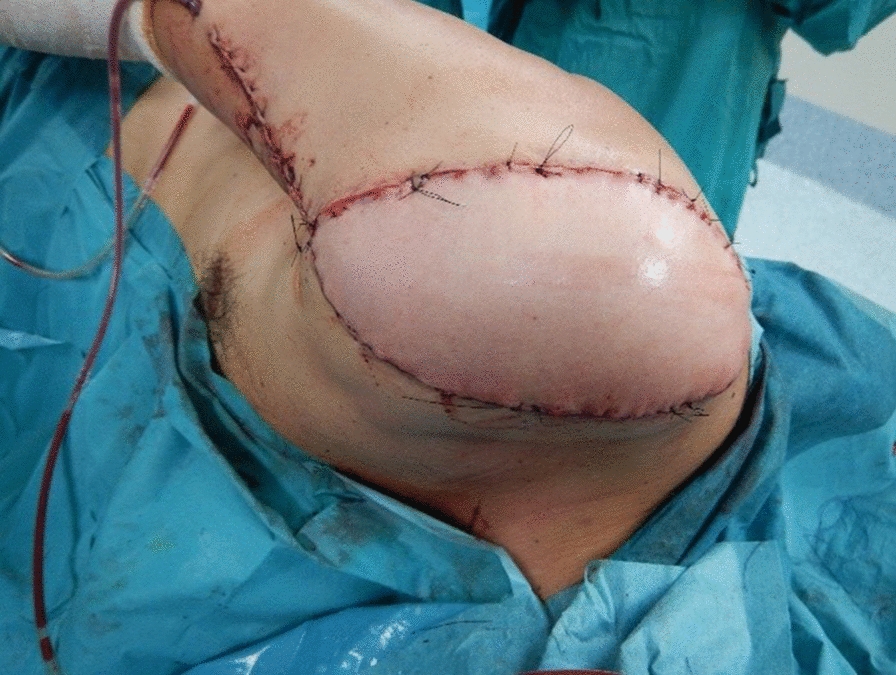
Postoperative view at the end of surgery. An homolateral pediculate latissimus dorsi myocutaneous flap was used to cover the defect

In July 2018, a wide surgical resection including the right anterior deltoid muscle and the lateral margin of the right pectoralis major muscle was performed, with a planned macroscopic margin of minimum 3 cm and the complete excision of the scar and the field of the previous surgery. The defect was reconstructed by right latissimus dorsi myocutaneous flap and polypropylene prosthesis (Figs. 3 and 4).

**Fig. 5:**
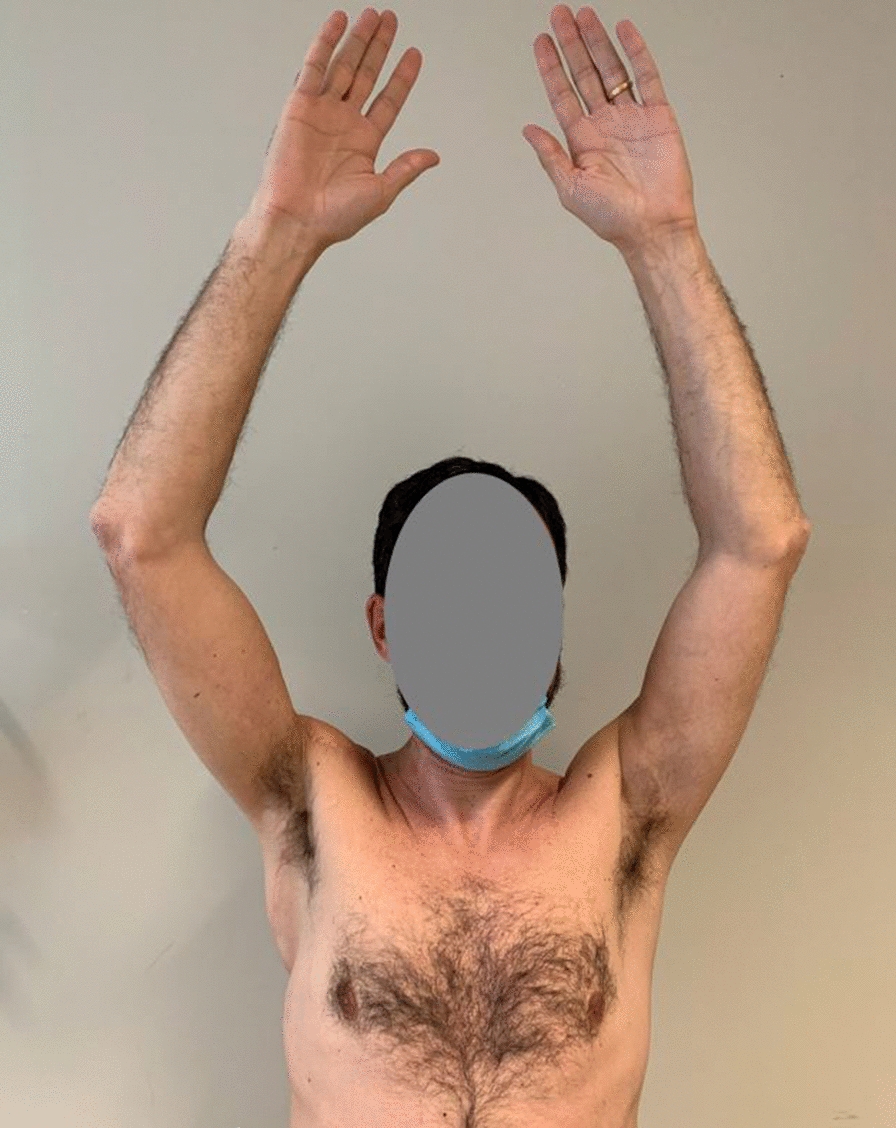
Patient at 24 months follow up

**Fig. 6:**
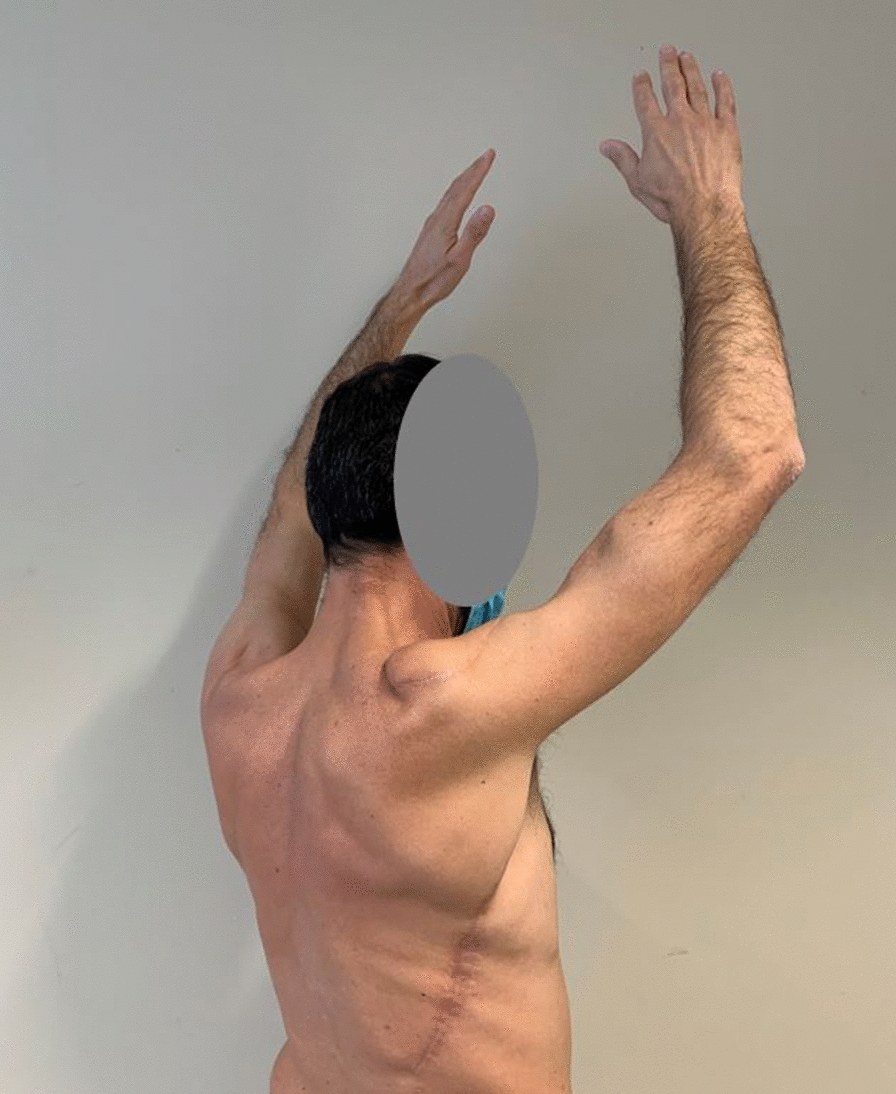
Patient at 24 months follow up

The size of the excised specimen was 23.5 × 10 × 3.5. Pathological examination was negative for viable tumor cells, showing only a fibrous subcutaneous scar. The patient had a complete response to preoperative RT. After MTB discussion, no further adjuvant treatments were provided. The patient did not show postoperative complications and was discharged on postoperative day 5. Neither relapse or systemic progression occurred in 24-months post-operative follow up. After the surgery the patient wore a shoulder brace for 15 days to avoid damage to the flap. Then a rehabilitation program consisting of 24 sessions (three times a week, lasting 1 h a session) was performed to improve the range of motion of the shoulder and motor performance of the shoulder girdle. No functional deficits were observed 6 months after surgery and at the 24-month follow up. Currently, after 2 years from surgery, at the clinical evaluation, the patient does not show differences in the flexion and abduction of the shoulder between right and left side (Fig. 5 and 6) and he confirms that he is leading a normal life without experiencing functional deficit or limitations in his daily life and his professional activities.

## Discussion

EMC is a rare subtype of soft tissue sarcoma (STS) showing indolence course but with a strong tendency to local recurrence and distant metastasis [1–5]. In the reported case, the patient, after inadeguate surgery in a non-referral center, was treated with a “watch and wait” approach by our sarcoma multidisciplinary tumor board according to literature data. Currently, immediate re-excision is justified when a macroscopic residual tumor is observed or when surgical or pathological reports identify piecemeal resection [14]. It is now demonstrated that re-excision (RE) delayed at the time of LR did not impair metastatic relapse-free survival (MRFS), or overall survival (OS) [14, 18–22]. Moreover, RE often requires a larger surgery that could sometimes be mutilating, sacrificing major motor function to warrant wide margin.

In the reported case, facing a confirmed deep-sited local recurrence after inadeguate surgical excision and suspected oncological contamination of the surgical field, RT followd by wild surgical excision were recommended according to ESMO-EURACAN Clinical practice guidelines [6].

Because of the delicate position of the mass and also taking into account the anamnesis of the patient—who is a young, active adult, working as an airplane pilot—our MTB decided to administer RT in neoadjuvant settings to reduce the irradiation field (and so the radio-exposition of the joints of the shoulder) and the long-term morbidity.

We found an uncommon dramatic radiological response after radiotherapy but strong literature data supporting a superiority of an exclusive radiotherapy instead of a combination of surgery and radiotherapy were lacking. Our MTB therefore decided to go with a wide re-excision extended to the scar and to the field of the previous surgery in order to reduce the risk of local recurrence in this young patient with a long life expectancy –also because a new local recurrence could not be managed anymore neither with an additional radiotherapy nor with a conservative surgery We balanced this decision with the predicted limited functional impairment expected after the planned surgery and we shared the choice with the patient, after multidisciplinary discussion.

Consistent literature data supports the use of RT in combination with surgery, in both the adjuvant and the neoadjuvant setting. EMC patients who received surgery alone showed significantly higher local recurrence rates than patients treated with a combination of surgery and RT [23]. Retrospective data suggests a potential survival benefit for radiation therapy in patients with localized EMC [12] and a prolonged local control for unrespectable patients [24]. Therefore literature data seems to suggest a central role for RT, which could routinely be added to surgical resection in order to obtain a better local control for this specific subtype of STS. Despite the well-documented results of RT with EMC, a complete pathological response to preoperative RT, such as those we observed, to our knowledge has never been reported in the literature.

It is interesting to note that, among all STS subtypes which are basically radioresistant, one of the most radiosensitive is the myxoid liposarcoma (MLS) [25, 26]. MLS sensibility to radiotherapy has been related to the tumor histological specific feature, characterized by the presence of a dense vascular pattern—radiation induced vascular damage determine hypoxia in the mass. Moreover, even in non-MLS STS, those with histological features similar to MLS have been shown to have a major pathological response after radiotherapy [27]. Even if EMC does not usually show such a vessel rich stroma [28], it seems to be a promising study field to correlate the histological feature of this tumor to its sensitivity to radiation therapy, comparing pre- and post- radiation specimens.

## Conclusions

In EMC, as in all STS, an optimal treatment strategy is crucial to guarantee the best outcomes for the patients. Patients evaluation in referral centers is mandatory to ensure accurate diagnosis and management. A multidisciplinary approach targeted on the single case characteristics of the specific case is the cornerstone to obtain the best oncologic and functional outcome for the patient. In agreement with literature data, our histopathological findings suggest RT to be promising to achieve the highest chance for local control and prolonged survival, especially in EMC patients. Further studies are needed to confirm if the improved local control guaranteed by combined RT and wide surgical excision will reflect also in a consistent survival benefit for EMC patients and if it is possible to identify which are the patients who could have the most benefits from radiotherapy.

## Data Availability

Not applicable.

## Abbreviations

EMC: Extraskeletal myxoid chondrosarcoma
STS: Soft tissue sarcoma
SRCs: Sarcoma referral centers
MRI: Magnetic Resonance Imaging
CT: Computed Tomography
RT: Radiotherapy
CHT: Chemotherapy
MTB: Multidisciplinary tumor board
LR: Local recurrence
RE: Re-excision
MRFS: Relapse-free survival
OS: Or overall survival
MLS: Myxoid liposarcoma
